# Intra-operative biopsy in chronic sinusitis detects pathogenic *Escherichia coli* that carry *fimG/H*, *fyuA* and *agn43* genes coding biofilm formation

**DOI:** 10.1371/journal.pone.0192899

**Published:** 2018-03-23

**Authors:** Michał Michalik, Alfred Samet, Andrzej Marszałek, Beata Krawczyk, Roman Kotłowski, Alex Nowicki, Tomasz Anyszek, Stella Nowicki, Józef Kur, Bogdan Nowicki

**Affiliations:** 1 Medical Center MML, Warsaw, Poland; 2 Medical Laboratories, SYNEVO, Warszawa, Poland; 3 Department of Molecular Biotechnology and Microbiology, Faculty of Chemistry, Gdańsk University of Technology, Gdańsk, Poland; 4 Nowicki Institute for Women’s Health Research (Now I for HeR), 114 Governors Way, Brentwood, TN, United States of America; University of Alabama at Birmingham, UNITED STATES

## Abstract

The aim of this study was to investigate whether or not surgical biopsy of sinus tissue in chronic sinusitis, not responsive to treatment, would detect *E*. *coli*. We intended to evaluate *E*. *coli* virulence genes, therefore dispute the causal role of such an unusual microorganism in chronic sinusitis, as well as consider effective pathogen-targeted therapy. Patients with *E*. *coli* isolated by intra-operative puncture biopsy were included in the study. Genetic analysis of *E*. *coli* isolates, including phylogenetic grouping and virulence factor characteristics, were done by multiplex PCR. We identified 26 patients with chronic sinusitis, in which 26 *E*. *coli* isolates were cultured. The *E*. *coli* isolates belonged mainly to pathogenic phylogenetic group B2, and carried multiple virulence genes. Three genes in particular were present in all (100%) of examined isolates, they were (1) marker *agn43* gene for forming biofilm, (2) type 1 fimbriae (*fimG/H* gene) and (3) yersiniabactin receptor (*fyuA*). Furthermore, a pseudo-phylogenetic tree of virulence genes distribution revealed possible cooperation between *agn43*, *fimG/H*, and *fyu*A in the coding of biofilm formation. Intra-operative-biopsy and culture-based therapy, targeting the isolated *E*. *coli*, coincided with long-term resolution of symptoms. This is the first report demonstrating an association between a highly pathogenic *E*. *coli*, chronic sinus infection, and resolution of symptoms upon *E*. *coli* targeted therapy, a significant finding due to the fact that *E*. *coli* has not been considered to be a commensal organism of the oropharynx or sinuses. We postulate that the simultaneous presence of three genes, each coding biofilm formation, may in part account for the chronicity of *E*. *coli* sinusitis.

## Introduction

Chronic sinusitis (CRS) is among the most frequent chronic conditions, affecting approximately 4 to 28% of the general public [1]. This disease is not only a socioeconomic burden on the community, but also significantly reduces the quality of life of those effected [1]. Chronic sinusitis is defined as an infection of the sinuses, which last for 12 weeks or more, a medical diagnosis which is made based on evidence of inflammation confirmed by rhinoscopy and imaging studies complemented by nasal and sinus bacterial cultures, plus allergy testing, and has a multifactorial etiology [2, 3, 4]. The management of chronic sinusitis involves a combination of antimicrobial treatment and surgery [2, 3] Although there is no direct evidence supporting genetic predisposition to CRS, this disease is more prevalent in patients with inherited mutations leading to disorders such as cystic fibrosis, Kartagener syndrome, allergies and asthma, many of which are direct causes of prolonged inflammation in CRS [4, 5, 6]. It has been described that during chronic sinusitis a malfunction of mucociliary clearance (MCC), epithelial barrier dysfunction, and deficiencies in the host immune response, lead to unresolved infection and inflammation directly resulting in tissue remodeling [6]. This tissue remodeling then further propagates the vicious cycle of deterioration and dysfunction of the sinuses’ natural defense mechanisms and yet another cycle of infection and mucosal injury. In addition, multiple indirect causes of chronic sinusitis have also been described. For example, patient ethnicity with further relation to socioeconomic status, geographic location, heritable components, and cultural factors in populations [6].

There are currently six widely accepted theories on chronic sinusitis etiology and pathogenesis ranging from microbial, or immunological in nature to the fault of signaling molecules generated by the metabolism of arachidonic acid [7]. Among the microbial etiologies, the most common culprits of sinusitis are *Staphylococcus aureus*, coagulase negative *Staphylococcus* sp., *Haemophilus influenza*, *Streptococcus pneumonia*, *Klebsiella pneumonia*, *Moraxella catharalis*, *and* less often, *Pseudomonas aeruginosa*, *Escherichia coli* or anaerobic species [8–14].

Further discussing the 6 theoretical etiologies of CRS, in addition to purely pathogenic bacterial and fungal causes of sinusitis one theory is that, changes in the host microbiome, may alter the normal microbe-host dialogue in humans, leading to the pathogenesis of CRS [15]. The microbiologic features of causative organisms in chronic sinusitis are not well established, and vary from Gram-positive if collected by swab to Gram-negative, including *E*. *coli*, if collected by biopsy [16]. Antibiotic therapy targeting pathogens classically implicated in sinusitis, while in the presence of Gram-negative pathogens, could augment the risk of therapeutic failure and/or chronic process through natural selection [17]. Yet another of the 6 common theories on CRS etiology is that, bacterial pathogenesis as the primary role in chronic sinusitis is in itself questionable, and rather bacterial virulence is considered a secondary cause of inflammation due to mucosal exposure to bacterial super-antigens [18]. So far the presence of *E*. *coli* in chronic sinusitis is poorly documented in literature, and has not been shown to be a commensal organism of the oropharynx or sinus cavities. The nature of *E*. *coli* virulence within a host suffering from chronic sinusitis remains largely unknown. There are only speculations about the role of biofilm [19–22] in the maintenance of inflammation in chronic sinusitis as documented for other diseases like *E*. *coli* gastrointestinal (GI) [23] and urinary tract infection (UTI) [24, 25]. It is thought, however, that biofilm formation is not singularly responsible for chronic sinusitis, but that correlation with other etiopathogenetic factors is necessary for the development of disease [1, 26]. While the role of virulence genes in chronic/recurrent GI and UT infection has been investigated, there is no such data available for chronic sinusitis [27–30, 8]. The aim of this study was to investigate whether surgical biopsy of sinus tissue in chronic sinusitis, not responsive to antibiotic treatment, would detect *E*. *coli*, and to evaluate *E*. *coli* virulence genes, therefore dispute the unlikely pathogenic role of such an unusual microorganism in chronic sinusitis.

## Methods

### Study population

The study, conducted in the Medical Center MML in Warsaw, in the years 2010–2014, was approved by the Bioethics Committee at the Medical University of Lodz RNN / 128/17 / EC dated 11.04.2017.”Research experiment design: *Escherichia coli* in the pathogenesis of chronic paranasal inflammation. Detection of virulence genes and evaluation of microbial susceptibility of highly pathogenic microorganisms from patients with chronic paranasal inflammation”. Patients were informed about their participation in the study and sign a written consent form. Patients' ages ranged from 20 to 63 years (mean age, 41 years 6 months), 18 of which were male and 8 female. Chronic sinusitis was diagnosed on the basis of the patient’s medical history and physical examination. The criteria established by the American Academy of Otolaryngology Head & Neck Surgery—pain or tightness in the area of the sinuses, retention of secretions in the sinuses and its difficult outflow and purulent discharge—were used to diagnose chronic sinusitis. Only chronic sinusitis patients, treated at the Medical Center MML in Warsaw, in whom *E*. *coli* was isolated were included in the study. The assessment of CRS in these patients was further confirmed by histopathology of the biopsied sinus mucosal lining, endoscopy and computed tomography.

### Isolation and identification of *E*. *coli*

Intra-operative puncture biopsy of the patient’s sinuses provided materials from which *E*. *coli* were isolated. Biopsies were transported to the microbiology lab within no longer than three hours from time of collection. Bacterial isolations and identification were performed according to bioMerieux procedures. The semiautomatic bioMerieux Vitek2 analyser was used for *E*. *coli* identification and antibiotic resistance. *E*. *coli* strains were stored at -80°C for further analysis.

### Genetic examination of *E*. *coli* isolates

Genomic DNA was isolated from individual bacterial colonies using a commercial kit (ExtractMe DNA bacteria Kit, BLIRT SA, Poland). Genotyping of strains by PCR MP (the polymerase chain reaction melting profiles) was carried out according to the procedure described by Krawczyk et al. [31]. The attribution of *E*. *coli* strains to a phylogenetic group was done by the multiplex PCR method as described by Clermont et al. [32].

The three multiplex PCRs were used for 12 virulence genes as a homemade test and two genes were separately amplified as a simplex. In multiplex PCR system no. I, six genes coding for virulence factors were detected: (*pap*C, *sfa*D/E, *fim*G/H coding the fimbriae P, S, and type 1 respectively, *usp* gene coding for uropathogen specific protein Usp, *cnf*1 gene coding cytotoxic necrotising factor 1, and *hly*A gene responsible for the production of hemolysin toxin. These virulence factors are important for the extra-intestinal strains of *E*. *coli* (EXPEC) especially for uropatogenic (UPEC) *E*. *coli* strains. Reaction conditions were as described by Adamus-Bialek et al., [33] with minor modifications by using DNA polymerase Hypernova BLIRT SA, POLAND.

In multiplex PCR system no. II, the sequence of three fragment genes, *kspMTII* [34] (synthesis capsule, group II), *iha* [35] (enterobactin Iha iron regulated gene homologue adhesion) and *focG* [34] (F1C fimbriae), were amplified. In multiplex PCR system III, the sequence of three fragment genes, *iutA* [36] (aerobactin receptor), *fyuA* [37,38] (yersiniabactin receptor) and *ibeA* [39] (invasion of brain endothelium A), were amplified.

In a separate PCR assays, *dra*CD (Dr fimbriae) [40, 41] and *agn*43 (outer membrane protein Agn43) [42] fragment genes were detected.

### Statistical analysis

Statistical analysis between phylogenetic groups B1 and B2 and virulence factors of *E*. *coli* was performed using the Fisher’s exact test. The results were interpreted based on a threshold of statistical significance set at *P* = 0.05. Free Statistics Software, Office for Research Development and Education, version 1.2.1, URL was used, (http://vassarstats.net/tab2x2.html).

#### Pseudophylogenetic tree

The Pseudo-phylogenetic tree was created based on gene distribution in *E*. *coli* isolates. The values corresponding to the lengths of the branches, stated as R-relation parameters, indicate the degree of relation between virulence genes. The cut-off values for R parameters are: a level close to R = 0 indicates an almost identical distribution of virulence genes, R = 0.5 denotes a relation of virulence genes distribution, and a total value close to R = 1 means unrelated distribution of virulence genes in tested isolates [43].

## Results

### Clinical evaluation

Study patients had a history of chronic sinusitis ranging from 1 to 4 years. During this period, the study group patients had been given from 3 to 9 different antibiotics (namely beta lactam antibiotics, macrolides and clindamycin) along with anywhere from 2 to 4 different laryngological treatments, which resulted in only short-term alleviation of symptoms, and no long-term relief. Intra-operative puncture biopsy detected *E*. *coli* in 26 chronic sinusitis patients. We did not perform culture of the oral cavity or sinus before surgery. Our main target was intraoperative surgery and culture of biopsy material. The culture results of 26 biopsy homogenates were all positive for *E*. *coli*, and showed both mono-and poly-microbial infections. The bacterial species that were co-cultured in different combinations with *E*. *coli* were found in the following number of cultures out of 26: *S*. *aureus*-17, *S*. *epidermidis*-12, *E*. *faecalis*-6, *K*. *oxytoca*-3, *P*. *aeruginosa*-3, *S*. *sanguinis*-2 *P*. *mirabilis*-1, *E*. *cloaceae*-1, *S*. *haemolyticus*-1. Of 26 biopsies, 3 patients had monoculture of *E*. *coli*. The cultures of 18 biopsies showed 2 pathogens, the majority of which were represented by *E*. *coli* plus one Gram (+) or Gram (–) pathogen. For example: *E*. *coli* plus *S*. *aureus* or *S*. *epidermidis*, *E*. *coli* plus *Enterococcus* or *E*. *coli* plus *Enterobacter* or *Klebsiella*. Five biopsies were multi-microbial and contained *E*. *coli* plus 3 or more pathogens. For example: *E*. *coli* plus *Proteus*, *Enterococcus*, and *S*. *aureus*. Due to scarcity of collected tissues we do not have Gram stain data. However, we did perform histopathology of the selected biopsies. An example of such data is as follows: Foci of sub-endothelial tissue edema with extensive infiltration with multiple plasma cells, plus 40% eosinophils and small number of granulocytes. This data is consistent with a chronic inflammatory process within the sinus tissue biopsy from which *E*. *coli* and or *E*. *coli* pus other microorganisms were isolated.

All 26 patients with *E*. *coli* fulfilled at least 3 of the 4 criteria of chronic sinusitis, as defined by the American Academy of Otolaryngology Head & Neck Surgery. In addition, in all patients with *E*. *coli* endoscopic examination showed features of chronic sinusitis. All patients received combination therapy: 1) Functional Endoscopic Sinus Surgery (FESS) in conjunction with (depending on the identified anatomical defects): correction of nasal turbinates by Celon method, rinsing the sinuses by Hydrodebrider method and 2) Anti-*E*. *coli* therapies taking into account the antibiotic sensitivity and the pharmacokinetic parameters of antibiotics including good penetration into the site of infection. Upon performance of culture and sensitivity, 23 *E*. *coli* isolates were found sensitive to Augmentin, and these patients responded to a combined treatment of surgery plus Augmentin. These 23 patients received a therapy of Augmentin 1000mg BID x 14 days. The remaining 3 patients were found to have *E*. *coli* isolates sensitive to Ciprofloxacin, and where treated accordingly. After completion of combination FESS plus targeted antibiotic therapy, laryngological examinations showed no signs of chronic sinusitis. Follow-up cultures using both swabs for colonization of the nose and throat, as well as sinuses aspirates were *E*. *coli* negative, for all the patients. The observation period after treatment was 3–16 months, within which all patients remained asymptomatic. Due to resolution of symptoms and negative cultures, a post treatment follow-up biopsy of sinus mucosa was not performed.

### Genotyping and phylogenetic group

Population genetics analyses of examined *E*. *coli* isolates by the PCR MP method showed a high level of genetic diversity. Each determined PCR MP genotype was represented only by one PCR MP pattern (data not shown). On the other hand, of the 26 analyzed isolates of *E*. *coli*, only 6 belonged to commensal phylogenetic group B1, and 20 (77%) belonged to the highly virulent phylogenetic group B2, (Table 1).

**Table 1:** Analysis of the phylogenetic group determination and the profile of virulence factors among the *E*. *coli* strains isolated from patients with chronic inflammation of the maxillary sinuses.

No.	Phylogenetic group	Virulence factor													
		*papC*	*sfa*	*fimG/H*	*afa/dr*	*cnf1*	*usp*	*hlyA*	*kspMTII*	*iha*	*focG*	*iutA*	*fyuA*	*ibeA*	*agn43*
**1**	B1	-	-	+	-	-	-	-	-	+	-	+	+	+	+
**2**	B1	-	-	+	-	-	-	-	-	+	-	+	+	+	+
**3**	B1	-	-	+	-	-	-	-	-	+	-	+	+	+	+
**4**	B1	-	+	+	-	+	-	+	-	+	-	+	+	-	+
**5**	B1	-	+	+	-	+	+	+	-	+	-	+	+	-	+
**6**	B1	-	+	+	-	+	+	-	-	-	-	+	+	+	+
**7**	B2	-	+	+	-	+	+	+	-	+	-	-	+	-	+
**8**	B2	+	+	+	-	+	+	+	-	+	-	-	+	+	+
**9**	B2	+	-	+	-	+	-	+	+	+	-	+	+	+	+
**10**	B2	+	+	+	-	+	+	+	-	+	-	-	+	+	+
**11**	B2	+	-	+	-	+	-	+	+	+	-	+	+	-	+
**12**	B2	-	-	+	-	-	+	-	+	-	+	+	+	-	+
**13**	B2	-	-	+	-	-	-	-	-	-	-	+	+	-	+
**14**	B2	+	+	+	-	+	+	+	+	-	-	-	+	+	+
**15**	B2	+	+	+	-	-	+	+	-	+	+	+	+	-	+
**16**	B2	+	+	+	-	-	+	+	-	+	+	+	+	-	+
**17**	B2	+	+	+	-	-	+	+	-	-	-	-	+	-	+
**18**	B2	+	+	+	-	-	+	+	-	-	-	-	+	-	+
**19**	B2	-	-	+	-	+	+	-	+	+	-	-	+	+	+
**20**	B2	+	+	+	-	-	+	+	+	-	-	-	+	-	+
**21**	B2	+	+	+	-	-	+	+	-	-	+	-	+	+	+
**22**	B2	+	+	+	-	-	+	+	+	-	-	-	+	-	+
**23**	B2	-	-	+	-	+	+	+	-	-	+	-	+	-	+
**24**	B2	+	+	+	-	-	+	+	-	+	+	-	+	-	+
**25**	B2	+	+	+	-	+	+	+	-	-	-	-	+	-	+
**26**	B2	+	+	+	-	+	+	+	-	-	-	-	+	-	+
	*P* values	**0.0020**^**3**^	0.6278	1	1	0.6776	**0.0277**^**3**^	**0.0277**^**3**^	0.1456	0.1695	0.2803	**0.0040**^**2**^	1	**0.0198**^**2**^	1

*papC*—P fimbriae, *sfa*—S fimbriae (*sfaD/sfaE*), *fimG/H*—type 1 fimbriae (*fimG/fimH)*, *afa/dr*-Dr fimbriae (*afa/draB–C*), *cnf1*—cytotoxic necrotizing factor, *usp—*bacteriocin Usp, *hlyA—*haemolysin, *kspMTII*—protein responsible for capsule formation, *iha*—enterobactin (siderofor receptor and adherence factor), *focG*—F1C fimbriae, *iutA*—aerobactin receptor, *fyuA*—yersiniabactin receptor, *ibeA*—invasive protein, *agn43*—adhesin 43 (biofilm formation). The *P* values represents associations of toxin genes with B1^2^ and B2^3^ phylogenetic groups.

Pathogenic *E*. *coli* strains usually belong to phylogenetic groups B2 or D. They often carry a variety of virulence-associated genes (VAGs) located on pathogenicity islands (PAIs) [37]. Whereas the strains from phylogenetic group A and B1 are low-virulence and considered commensal [32, 40–43]. The results of our examination were not expected, as highly pathogenic *E*. *coli* would not be likely to cause chronic sinusitis in which obstructive features appear to play a key role.

### Prevalence of virulence factor genes

**Fig 1:**
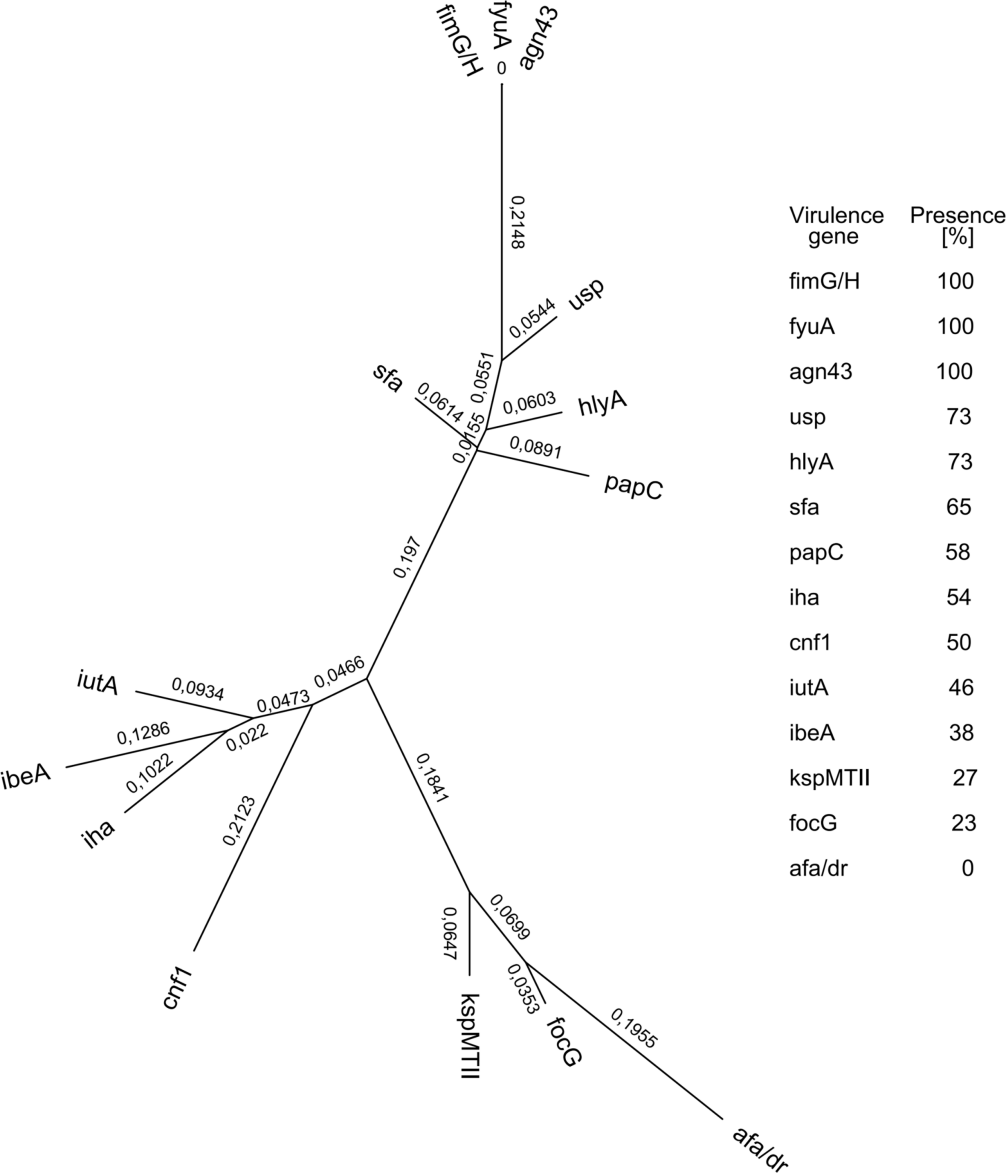
Pseudo-phylogenetic tree presenting virulence genes in *E*. *coli* strains isolated from patients with chronic inflammation of the maxillary sinuses. The values attributed to each branch (R-relation parameters) indicate the degree of correlation between the presence of virulence genes. A level close to R = 0 denotes almost identical virulence genes distribution, R = 0.5 means there is a relation between distribution of virulence genes, and a total value close to R = 1 reveals unrelated distribution of virulence genes in *E*. *coli* isolates.

The one-dimensional analysis (Table 1) and distribution similarity (Fig 1.) of virulence genes carried out on the pool of examined isolates from all 26 patients shows that the marker gene for forming biofilm *(agn43)*, type 1 fimbriae gene (*fimG/H*), and yersiniabactin receptor (*fyuA*) were the most frequently occurring virulence factor genes (100%) (26 out of 26 in each case).

The occurrence of the *usp*, *hlyA and sfa* genes (19, 19 and 17 out of 26, respectively) was 73%, 73%, and 65%, respectively. Cytotoxic necrotising factor 1 (*cnf1* gene) was detected in 13 out of 26 (50%) isolates, enterobactin Iha gene (*iha*) was detected in 14 out of 26 strains (54%) and aerobactin receptor (*iutA*) in 12 out of 26 strains (46%). Only seven of the isolates tested had the capsule *kspMTII* gene. There was an absence of Dr fimbriae coding *draC–D* genes characteristic for uropathogenic *E*. *coli*, which causes recurrent UTI and interstitial renal invasion. Consistent with the predicted virulence of group B2, the occurrence of the *pap*C—encoding P fimbriae, *sfa—*encoding S fimbriae, *hlyA—*coding for hemolysin and, *usp* genes—encoding bacteriocin Usp (15, 14, 17 and 17 out of 20, respectively) were statistically significant in phylogenetic group B2 (P values: 0,0020; 0,0277; 0,0277, respectively), while *ibeA* and *iutA* genes were statistically significant in phylogenetic group B1 (Table 1).

The numbers (R-relation parameters) indicate the degree of relatedness of the virulence genes. A level close to R = 0 denotes almost identical virulence genes distribution. Moreover, pseudo-phylogenetic analysis of virulence factors that are present in patients at the level of ≥50% allows for mathematical inference of cooperation between selected genes in the development of the disease. For example, 58% to 73% of isolates, were positive for *usp*, *hlyA*, *sfa* and *papC* genes, demarcating a strong possibility of combined action in the pathogenesis of the disease, further upheld statistically by a relatively low relation parameter R = 0.2652 (Fig 1). Furthermore, cumulative R parameters between 7 genes (*fimG/H*, *fyuA*, *agn43*, *usp*, *hlyA*, *sfa* and *papC*) suggest that all of them could be involved in the pathogenesis of chronic sinusitis, because R values among them are all below 0.5 (Fig 1).

### Discussion

This is the first report demonstrating an association between a highly pathogenic *E*. *coli*, chronic sinus infection, and the resolution of symptoms upon *E*. *coli* targeted therapy. Although at first glance one might believe the results of this study to show a correlative relationship, we propose that, because this is a clinical translational study, correlation between a specific pathogen, genotype pattern, and clinical response to treatment, show that the study results are consistent with a causal relationship.

Our data supports the hypothesis that pathogenic *E*. *coli* can be detected in a set of patients with chronic sinusitis not responsive to therapy. Chronic infection of the sinuses in examined patients occurred with a non-random, pathogenic *E*. *coli* that carried bacterial biofilm and multiple virulence coding genes. Intra-operative-biopsy and culture-based therapy targeting the detected *E*. *coli* coincided with long-term resolution of symptoms.

The treatment of patients with chronic sinusitis is a daily challenge due to several factors that are responsible for the patho-physiology of this disease. Environmental factors include numerous microorganisms that colonize the mucous membranes of the oro-pharynx, which often mask the detection of the true etiologic factor responsible for the chronic sinusitis. Resolution of chronic sinusitis, following biopsy directed antibiotic therapy and FESS, suggested the contribution of *E*. *coli*, and perhaps its genetic make-up to be a direct causal factor of the pathogenesis of chronic sinusitis. Whether the presence of *E*. *coli* biofilm in patients with chronic sinusitis is responsible for poor results after surgical treatment with FESS only, remains to be investigated. We consider the hypothesis that anti-*E*. *coli* therapies did lead to the eradication of inflammation, because it did take into account the existence of a previously undetected pathogen.

There are limitations to this study that should be recognized. The present investigation was a study of healthcare–seeking adults, with only those included that were *E*. *coli* positive. Thus, the results may not apply to the rest of the population. Due to the resolution of symptoms and negative cultures, a post treatment follow-up biopsy of sinus mucosa was not performed. The prevalence of chronic sinusitis with *E*. *coli* vs. mixed infection (ie. *E*. *coli* combined with aerobes plus anaerobes) has not been well evaluated in the literature and will be a subject for our future study. Adjusting for potential risk factors, such as smoking status, is unable to be performed as a direct result of non-inclusion in the database.

The obtained results allow the theorization that there is genetic uniformity between strains of *E*. *coli* isolated from patients with chronic sinusitis. The correlation of virulent genotypes and the phylogenetic group B2 background for *E*. *coli* strains isolated from examined patients is consistent with the interpretation that chronic sinusitis was not caused by random, nonpathogenic colonizers, but rather by highly virulent *E*. *coli*. Although the gene profiles represented a great extent of similarity to extraintestinal-pathogenic *E*. *coli*, some features suggest that chronic sinusitis isolates could be unique to the sinus location [20, 25, 26]. In this context the *sfa* adhesin gene, which is associated with meningitis, could contribute to the pathogenesis of chronic sinusitis and meningitis via similar cytophysiological pathways, and further, histological similarities between target tissue based on anatomical adjacency of the CNS and para-nasal sinuses. *hly*A and *usp* genes encoding bacterial toxins were present in over 70% of the isolates, which indicates a high level virulence of these strains. Some virulent *E*. *coli*, due to its invasive and/or toxic/hemolytic activity may multiply in the cytoplasm of host cells and persist due to their capacity for iron acquisition, therefore contributing to the chronic process of infection [41, 38]. These factors are especially typical for a phylogenetic group B2, also in our study (*p* value. 0,0277 for each one). In contrast, lack of *dra*/*afa* adhesins involved in chronic/recurrent UTI and gestational pyelonephritis, stress a unique gene pattern of chronic sinusitis isolates and, perhaps more specifically, sinus tropism [25, 26].

Unexpectedly, 100% of *E*. *coli* isolated from examined patients carried *agn43*, *fimG/H* and *fyu*A genes, all of which are implicated in UTI. A pseudo-phylogenetic tree of virulence genes distribution revealed possible cooperation between *agn43*, *fimG/H and fyu*A [38, 44, 45]. These findings are significant because of their roles in biofilm formation: *agn43* contributes to *E*. *coli-E*. *coli* self-binding, *fim* genes, coding for type 1 fimbriae, allow for *E*. *coli* aggregation and adherence to mannose receptors on mucosal membranes, and the yersiniabactin receptor *fyuA* contributes to biofilm formation and iron scavenging, a virulence factor implicated in uro-septicemia. The key question remains, could these three genes, each of which independently contribute to biofim formation, work in concert—enhancing the process of biofilm development leading to the creation of a “super”-biofilm? Such a bacterial barrier would outsmart the host, escape immune attack, and block penetration of antibiotics; creating resistant pathogens, protracted colonization, and chronic inflammation of the sinuses. In the future, we plan on full genome analysis of *E*. *coli* isolates, to further our understanding of virulence factors and threat level. Furthermore, experimental research and clinical trial may be necessary to evaluate strategies to disrupt the barrier formed by this biofilm. Based on our study we recommend that all patients with chronic sinusitis should be considered for intraoperative biopsy. Obtained samples should be homogenized and cultured for *E*. *coli*, and other potential pathogens, therefore allowing targeted therapy. We also suggest consideration of further genotyping of *E*. *coli* to assure that the isolated *E*. *coli* represent features of a virulent pathogen.

In the future, use of vaccines and attachment blockers might be an effective therapeutic consideration as the rapid spread of virulent, multi-antibiotic resistant *E*. *coli* becomes an emerging global health threat. For example, vaccines such as, anti-iron binding protein and anti-type 1 fimbriae, in combination with *E*. *coli* adherence blockers, which have been tested on colonization/invasion assays in relation to UTI and /or uro-septicemia, could be tested in chronic sinusitis for their effect on disintegration of biofilm and eradication of pathogenic *E*. *coli* from the sinus cavities [46, 47].
